# CagI Is an Essential Component of the *Helicobacter pylori* Cag Type IV Secretion System and Forms a Complex with CagL

**DOI:** 10.1371/journal.pone.0035341

**Published:** 2012-04-06

**Authors:** Kieu Thuy Pham, Evelyn Weiss, Luisa F. Jiménez Soto, Ute Breithaupt, Rainer Haas, Wolfgang Fischer

**Affiliations:** Max von Pettenkofer-Institut für Hygiene und Medizinische Mikrobiologie, Ludwig-Maximilians-Universität, München, Germany; University of Hyderabad, India

## Abstract

*Helicobacter pylori*, the causative agent of type B gastritis, peptic ulcers, gastric adenocarcinoma and MALT lymphoma, uses the Cag type IV secretion system to induce a strong proinflammatory response in the gastric mucosa and to inject its effector protein CagA into gastric cells. CagA translocation results in altered host cell gene expression profiles and cytoskeletal rearrangements, and it is considered as a major bacterial virulence trait. Recently, it has been shown that binding of the type IV secretion apparatus to integrin receptors on target cells is a crucial step in the translocation process. Several bacterial proteins, including the Cag-specific components CagL and CagI, have been involved in this interaction. Here, we have examined the localization and interactions of CagI in the bacterial cell. Since the *cagI* gene overlaps and is co-transcribed with the *cagL* gene, the role of CagI for type IV secretion system function has been difficult to assess, and conflicting results have been reported regarding its involvement in the proinflammatory response. Using a marker-free gene deletion approach and genetic complementation, we show now that CagI is an essential component of the Cag type IV secretion apparatus for both CagA translocation and interleukin-8 induction. CagI is distributed over soluble and membrane-associated pools and seems to be partly surface-exposed. Deletion of several genes encoding essential Cag components has an impact on protein levels of CagI and CagL, suggesting that both proteins require partial assembly of the secretion apparatus. Finally, we show by co-immunoprecipitation that CagI and CagL interact with each other. Taken together, our results indicate that CagI and CagL form a functional complex which is formed at a late stage of secretion apparatus assembly.

## Introduction

The human gastric pathogen *H. pylori* is the principal cause of chronic active gastritis and peptic ulcer disease, and it is involved in development of gastric adenocarcinoma and mucosa-associated lymphoid tissue (MALT) lymphoma [1], [2]. The molecular mechanisms leading to development of ulcers or cancer are not well-understood, but both host and bacterial factors are likely to contribute to disease development [3]. One of the major bacterial virulence factors is the *cag* (*cytotoxin-associated gene*) pathogenicity island, a 37 kb genomic island present only in a subset of *H. pylori* isolates and clearly associated with an enhanced risk of developing peptic ulcers or adenocarcinoma. The *cag* pathogenicity island encodes the Cag type IV secretion system, which induces secretion of chemokines such as interleukin-8 (IL-8) from gastric epithelial cells and is thus responsible for a strong proinflammatory response during infection, and the effector protein CagA which is translocated into epithelial and potentially other host cells. Several cellular interaction partners of CagA and associated changes in cellular functions have been described, but its exact function in the infection process is not clear. However, due to its correlation with the risk of cancer development [4], its role in pathogenesis in a Mongolian gerbil infection model [5], and direct evidence from transgenic mice [6], CagA is often considered as a bacterial oncoprotein [7].

Similar to other type IV secretion systems, components of the Cag system assemble into a multiprotein secretion apparatus spanning both bacterial membranes [8]. Since the Cag system contains proteins with common features or sequence similarities to most or all essential components of prototypical type IV secretion systems, such as the VirB system of *Agrobacterium tumefaciens* or plasmid conjugation systems, it is believed that the structure of the secretion apparatus and its molecular mechanisms of action are analogous. This implies the presence of a core complex bridging the cytoplasmic and outer membranes [9], of pilus-like structures at the bacterial cell surface [10], [11], and of cytoplasmic or inner membrane-associated ATPases providing the energy for secretion apparatus assembly and protein transport. The pilus-like appendages on the bacterial surface seem to contain several apparatus proteins, but their exact composition has not been determined [10]–[12]. Interestingly, however, several proteins that have been shown or suspected to be part of these surface structures, interact with β1 integrins on the host cell surface. Proteins contributing to this interaction include the substrate CagA as well as the proteins CagL, CagY and CagI [13], [14]. Whereas CagY has a C-terminal domain with sequence similarity to VirB10 family proteins and CagL has been considered as a VirB5-like adhesin of the type IV secretion apparatus [15], CagI is a unique component without any sequence similarities to components of prototypical type IV systems, or to any other known protein. Although isogenic *cagI* mutants have been examined in several studies, there are conflicting reports about CagI requirement for type IV secretion system function. One study found that deletion of the *cagI* gene resulted in an impairment of CagA translocation, but not IL-8 induction [16], and two others found that the presence of *cagI* is required for IL-8 induction as well [17], [18]. Since *cagI* is part of an operon containing several other genes involved in type IV secretion [19], and since none of these studies included complementation of a *cagI* mutant, the actual contribution of CagI to these phenotypes is not clear. However, a recent study which included complementation of a *cagI* mutant provided direct evidence that CagI is required for IL-8 induction [20].

In this study, we have constructed a set of *cagI* mutants with the aim of avoiding polar effects on expression of the essential downstream gene *cagL*, and we have complemented the mutants to gain insight into CagI function. Infection experiments show that CagI is an essential component of the Cag type IV secretion apparatus, required for both IL-8 induction and CagA translocation. We provide evidence that CagI is, at least partly, associated with the bacterial outer membrane, and exposed to the bacterial surface. Moreover, we show that it interacts with the putative integrin ligand CagL. These data suggest that both proteins may form a complex at the bacterial cell surface, and that this complex is required for productive interactions with target cells.

## Results

### Deletion of the *CagI* gene Results in Low CagL Levels

**Figure 1:**
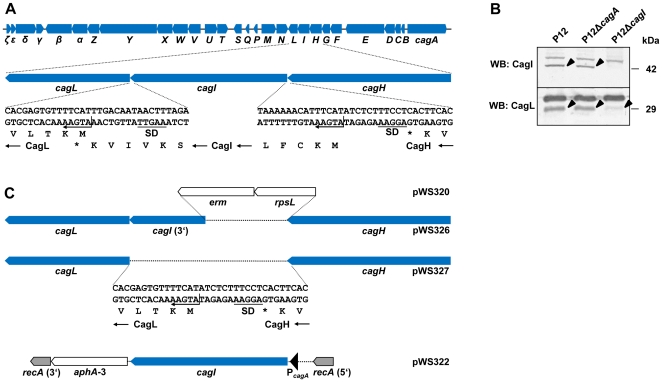
Operon organization of the *cagH*, *cagI* and *cagL* genes. (A) Gene arrangement of the *cag* pathogenicity island in *H. pylori* strain P12. Genes are represented as arrows, and their designations are indicated below. The operon containing *cagI* may comprise five (*cagF*, *cagG*, *cagH*, *cagI* and *cagL*) or even more genes, but *cagH*, *cagI* and *cagL* are particularly tightly associated since they have overlapping reading frames or Shine-Dalgarno (SD) sequences. Translational starts are indicated by arrows. (B) A *cagI* deletion mutant containing a terminatorless chloramphenicol resistance cassette [16] was generated by transformation with plasmid pSO171. A polyclonal rabbit antiserum raised against CagI was used for immunoblot analysis of whole cell lysates of the P12 wild-type strain or isogenic *cagA* or *cagI* mutants. The same lysates were examined by Western blot for the presence of CagL using the polyclonal rabbit antiserum AK271. CagI and CagL protein bands, respectively, are marked by arrowheads. Note that both antisera recognize cross-reactive bands, but anti-CagI does not react with CagL or vice versa (data not shown). (C) Plasmid constructs used for generation and complementation of *cagI* mutants. A counterselection strategy was used for generating marker-free deletion mutants. Mutants generated with plasmids pWS320 and pWS326 retain the 3′ part of *cagI*, whereas the mutant generated with plasmid pWS327 has a complete deletion without any *cagI* traces. For complementation of *cagI* mutants *in trans*, the *cagI* gene was cloned under the control of the *cagA* promoter and integrated into the *recA* gene (plasmid pWS322).

There have been conflicting results concerning the requirement of CagI for induction of IL-8 secretion from gastric epithelial cells in previous studies [16]–[18]. One reason for this discrepancy might be differences in the *H. pylori* strains used in each study, but it might also be due to subtle differences in gene expression patterns in the respective *cagI* mutants, given that *cagI* is part of an operon structure with several alternative transcriptional start sites [19]. Closer inspection of the *cagI* locus shows that the 3′ end of *cagI* overlaps with the start codon of *cagL*, and the Shine-Dalgarno sequence of *cagI* overlaps with the stop codon of *cagH* (Fig. 1A). Thus, *cagI* deletion and replacement by a resistance gene cassette may have polar effects on expression of the *cagL* gene. We sought to re-evaluate the contribution of *cagI* for type IV secretion system function by constructing *cagI* mutants devoid of such polar effects. To check for production of the CagI protein, we raised an antiserum against a synthetic peptide corresponding to a C-terminal CagI region (amino acids 350–368; see Materials and Methods). This antiserum recognized a 42 kDa protein in immunoblots from whole cell lysates of strain P12 or its isogenic *cagA* mutant, but not from lysates of an isogenic P12 *cagI* mutant generated with the deletion plasmid pSO171 (Table 1) (Fig. 1B). Control immunoblots with a CagL antiserum showed a strong reduction of CagL levels in the *cagI* mutant (Fig. 1B), suggesting that insertion of the chloramphenicol resistance cassette in the *cagI* locus exerted a downstream transcriptional effect. Alternatively, it is possible that the absence of the CagI protein influences CagL production and/or stability.

**Table 1:** Strains and plasmids used in this study.

Strain/Plasmid	Description	Source/Reference
P12	wild-type strain	[34]
P12Δ*cagδ*	P12 transformed with plasmid pWS145 [16]	This study
P12Δ*cagγ*	P12 transformed with plasmid pJP94 [16]	This study
P12Δ*cagβ*	P12 transformed with plasmid pJP93a [16]	[25]
P12Δ*cagα*	P12 transformed with plasmid pRB24 [16]	This study
P12Δ*cagZ*	P12 transformed with plasmid pRB25 [16]	[25]
P12Δ*cagY* pJP89a	P12 transformed with plasmid pJP89a [16]	This study
P12Δ*cagX*	P12 transformed with plasmid pJP90 [16]	This study
P12Δ*cagW*	P12 transformed with plasmid pRB22 [16]	This study
P12Δ*cagV*	P12 transformed with plasmid pRB26 [16]	This study
P12Δ*cagU*	P12 transformed with plasmid pWS139 [16]	This study
P12Δ*cagT*	P12 transformed with plasmid pJP95 [16]	This study
P12Δ*cagM*	P12 transformed with plasmid pBG11 [16]	This study
P12Δ*cagN*	P12 transformed with plasmid pBG10 [16]	This study
P12Δ*cagL*	P12 transformed with plasmid pWS290	[14]
P12Δ*cagI*	P12 transformed with plasmid pSO171 [16]	This study
P12Δ*cagH*	P12 transformed with plasmid pSO172 [16]	This study
P12Δ*cagG*	P12 transformed with plasmid pSO173 [16]	This study
P12Δ*cagF*	P12 transformed with plasmid pSO174 [16]	[26]
P12Δ*cagE*	P12 transformed with plasmid pBG17 [16]	This study
P12Δ*cagD*	P12 transformed with plasmid pSO176 [16]	This study
P12Δ*cagC*	P12 transformed with plasmid pBG16 [16]	This study
P12Δ*cagA*	P12 transformed with plasmid pJP52 [16]	[33]
pWS320	*cagI* (5′) deletion plasmid; *rpsL-erm* insertion	This study
pWS322	*recA* insertion plasmid; *cagI* behind *cagA* promoter	This study
pWS325	*cagI* (5′) deletion plasmid; *cat* insertion	This study
pWS326	*cagI* (5′) deletion plasmid (marker-free)	This study
pWS327	*cagI* deletion plasmid (marker-free)	This study

**Figure 2:**
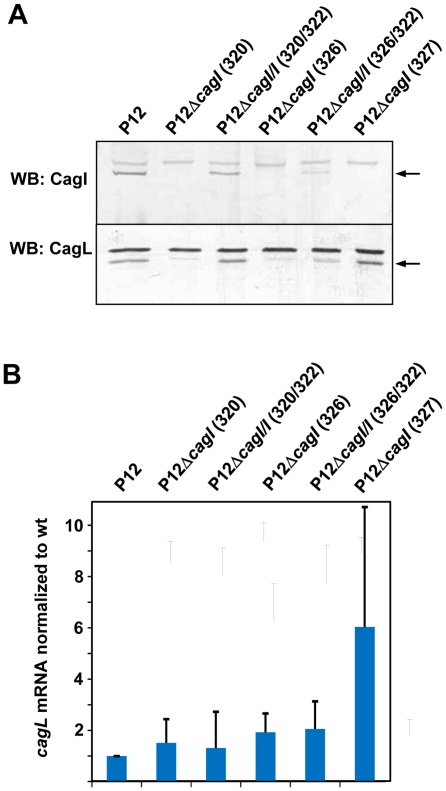
Analysis of CagI and CagL production, and of *cagL* transcription, in *cagI* mutants and complemented strains. (A) Whole cell lysates of the P12 wild-type strain and the indicated *cagI* mutants or complemented mutants were examined for CagI and CagL production by immunoblot analysis. Numbers in brackets indicate the plasmids used for mutant generation or complementation. (B) The indicated strains were grown in liquid culture to mid-exponential phase, and harvested for total RNA preparation and cDNA synthesis. Transcript levels of *cagL* were analyzed by quantitative real-time PCR (qPCR) and normalized to levels of 16S-rRNA for each cDNA sample. Bars represent mean values and standard deviations of *cagL* transcript levels in relation to wild-type levels for at least three independent RNA preparations.

Since CagL is essential for both CagA translocation and induction of IL-8 secretion [16], the role of CagI for type IV secretion system function could thus not unambiguously be demonstrated with this mutant. To overcome this problem, we used a marker-free deletion procedure involving the streptomycin counter-selection system [21] to construct *cagI* deletion mutants. Since an alternative transcriptional start site for *cagL* has been identified in the 3′ region of the *cagI* gene [19], we constructed mutants in strain P12 in which the 5′ *cagI* part was either replaced by a streptomycin sensitivity/erythromycin resistance cassette (using plasmid pWS320), or deleted without insertion of a resistance marker (using plasmid pWS326; Fig. 1C). Western blot analysis with the polyclonal CagI antiserum demonstrated that CagI production was lost in these mutants, as expected, but *cagI* deletion again resulted in strongly reduced CagL protein levels (Fig. 2A, lanes 2 and 4). Complementation of the *cagI* mutants with a chromosomal (*recA*) integration vector containing the *cagI* gene under the control of the *cagA* promoter (plasmid pWS322; Fig. 1C) resulted not only in CagI production, but also in restored CagL production (Fig. 2A; lanes 3 and 5). This suggested that the reduced CagL levels observed in the *cagI* mutants were not caused by transcriptional effects of resistance gene cassette insertion or gene deletion, but rather by direct effects of CagI on CagL.

To corroborate this conclusion, we sought to quantify *cagL* transcript levels in these mutants. For this purpose, we prepared total RNA from liquid cultures of the different *H. pylori* strains, carried out reverse transcription into cDNA, and performed conventional as well as real-time PCR with *cagL*-specific primers and control primers specific for 16S-rRNA. In control PCRs, we obtained *cagL*-specific bands from cDNA, but not mRNA from P12 wild-type bacteria, confirming that the RNA preparation was not contaminated by genomic DNA (data not shown). Transcript quantification by qPCR showed no significant difference in *cagL* levels between wild-type bacteria and each *cagI* mutant (Fig. 2B), demonstrating that deletion of the 5′ *cagI* region did not interfere with transcription of the *cagL* gene.

### CagI is Required for CagA Translocation and IL-8 Induction

**Figure 3:**
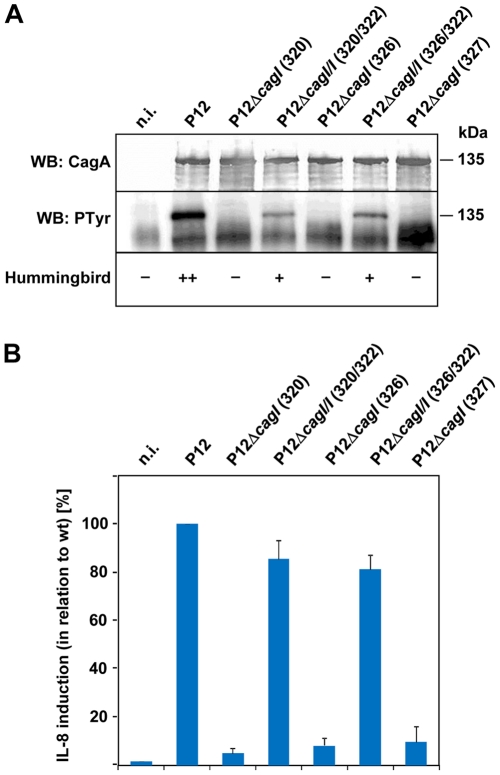
CagI is an essential component of the Cag type IV secretion apparatus. (A) AGS cells were infected for 4 h with the indicated strains at a multiplicity of infection of 100, and infection lysates were examined by immunoblot for the presence of CagA and CagA tyrosine phosphorylation (PTyr). Infected cells were also examined by light microscopy for induction of the hummingbird phenotype. (B) Culture supernatants of AGS cells infected with the indicated *H. pylori* strains as above were tested for their IL-8 content. IL-8 values induced by wild-type P12 were set to 100%, and other values are given in relation to wild-type levels. Data shown are average values of at least three independent experiments with standard deviations.

To examine the impact of *cagI* deletion on functionality of the Cag type IV secretion system, we performed infection experiments of AGS epithelial cells with *H. pylori* for 4 hours, and determined tyrosine phosphorylation of the CagA protein by Western blot analysis and induction of IL-8 secretion by sandwich ELISA. Infection experiments with the wild-type strain and the pWS320 and pWS326 mutants showed that *cagI* deletion results in a CagA translocation deficiency, as shown previously (Fig. 3A). In addition, these *cagI* mutants were unable to induce IL-8 secretion from AGS cells (Fig. 3B). Complementation of these mutants restored both CagA translocation and IL-8 induction after AGS cell infection (Figs. 3A and 3B).

While these results indicated an involvement of CagI in both type IV secretion-associated phenotypes, it could still not be ruled out that the differing CagL levels are in fact responsible for these phenotypes. Therefore, we complemented the pWS320 *cagI* mutant with a chromosomal integration vector harboring the *cagL* gene under the control of the *cagA* promoter. The resulting strain produced an intermediate amount of CagL, but was nevertheless defective for CagA translocation and IL-8 induction (data not shown), suggesting that CagI is independently required for type IV secretion system function. Furthermore, we generated a marker-free *cagI* deletion mutant (using plasmid pWS327; Fig. 1C) in which *cagI* was deleted in such a way that the *cagI* Shine-Dalgarno sequence is used as a ribosomal binding site for *cagL*, providing a “bypass” in the operon from *cagH* directly to *cagL*. Surprisingly, this *cagI* mutant produced normal levels of CagL (Fig. 2A), indicating that CagI is not absolutely required for CagL production or stability. However, quantification of *cagL* transcripts by qPCR showed a 6.04 ± 4.67-fold increase of *cagL* mRNA levels in the pWS327 mutant in comparison to wild-type bacteria (Fig. 2B), suggesting that precise deletion of the *cagI* gene resulted in a more effective transcription of the *cagL* gene, possibly via the *cagF* promoter. Interestingly, the *cagI* mutant obtained with plasmid pWS327 was also completely defective in both CagA translocation and IL-8 induction (Figs. 3A and 3B). Taken together, these results show that CagI is by itself an essential component of the Cag type IV secretion apparatus.

### CagI and CagL Protein Levels in *H. Pylori* Cells Depend on the Presence of Various Secretion Apparatus Components

**Figure 4:**
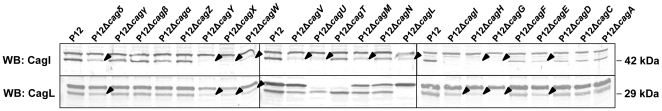
Presence of several Cag components influences CagI and CagL protein levels. Whole cell lysates of equal amounts of the wild-type strain P12 and of isogenic mutants in single *cag* genes (Table 1) were separated by SDS-PAGE and examined by immunoblotting with the anti-CagI and anti-CagL antisera, respectively. Representative immunoblots are shown. Note that the *cagI* mutant shown here was generated with plasmid pWS327. Arrowheads indicate the positions of CagI and CagL protein bands, respectively.

Mutual interdependence of protein levels is often found in multiprotein complexes such as type IV secretion machines, and has often been interpreted as stabilizing effects due to the presence of corresponding protein-protein interactions [22]–[25]. To determine if such effects occur between other Cag components and either CagI or CagL, we generated isogenic mutants of strain P12 in each *cag* gene known to have at least a partial function for Cag type IV secretion (Table 1), and we examined cell lysates of these mutants for CagI and CagL production by immunoblotting. As shown in Fig. 4, CagI production was influenced by the absence of several *cag* genes. Notably, the *cagX*, *cagY*, *cagH* and *cagG* mutants produced virtually no CagI, and several further mutants (Δ*cagδ*, Δ*cagW*, Δ*cagV*, Δ*cagU*, Δ*cagM*, Δ*cagL* and Δ*cagE*) produced significantly reduced amounts of CagI. In contrast, the *cagF* mutant produced normal levels of CagI. This suggests that inner membrane-associated structural components (CagU, CagV, CagW, CagY), components of a putative outer membrane-associated subcomplex (CagX, CagM, Cagδ), and also CagL, may have an impact on CagI stability. Interestingly, CagL levels were strongly reduced in the *cagY* and *cagX* mutants as well (Fig. 4), whereas control measurements by qPCR showed that the levels of *cagL* transcripts were not significantly different in these mutants (data not shown). Significant reductions of CagL levels were also found in the *cagδ*, *cagW*, *cagI*, *cagH*, *cagG* and *cagE* mutants, but not in other mutants showing reduced CagI levels (Δ*cagV*, Δ*cagU*, Δ*cagM*), and again not in the *cagF* mutant (Fig. 4).

### CagI is Present in Different Subcellular Pools and is Partly Surface-exposed

**Figure 5:**
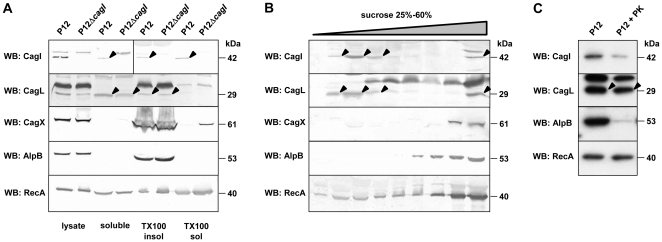
Localization of CagI and CagL in bacterial cell fractions. (A) *H. pylori* cells grown in liquid culture for 24–48 hours were lysed by ultrasonication and subsequently fractionated into soluble and insoluble proteins by ultracentrifugation. Ultracentrifugation pellets containing membrane-associated and other insoluble proteins were extracted with 1% triton X-100 to separate outer membrane-associated (TX100 insol) from inner membrane-associated (TX100 sol) proteins. Comparable amounts of each fraction were analysed by immunoblot for their CagI and CagL content. As controls, immunoblots against the outer membrane-associated proteins CagX and AlpB, and the partly soluble and partly inner membrane-associated protein RecA were used. Representative immunoblots are shown. CagI and CagL bands are indicated by arrowheads. (B) Ultracentrifugation pellets were resuspended and subjected to isopycnic density gradient centrifugation on 25–60% sucrose gradients. Fractions were collected from the gradients and analyzed by immunoblotting with the indicated antisera. CagI and CagL bands are indicated by arrowheads. (C) Bacteria were subjected to limited proteolytic digestion by proteinase K. Equal amounts of untreated control cells (P12) and proteinase K-treated bacteria (P12 PK) were analyzed by immunoblot with the indicated antisera.

To obtain evidence for CagI and CagL localization, we fractionated bacterial cells into soluble and membrane-associated components, and extracted the total membrane fraction with 1% triton X-100, which preferentially extracts cytoplasmic membrane proteins [26]. In these fractionations, CagI was found both in the membrane fraction and in the soluble fraction containing cytoplasmic and periplasmic proteins (Fig. 5A). Membrane-associated CagI was not completely extracted by triton X-100, suggesting that CagI might be partly associated with the outer membrane. In contrast, the majority of CagL was found in the soluble fraction, and only a minor part in the total membrane fraction from where it was not extracted by triton X-100 (Fig. 5A). The distribution of CagL in the fractions was not significantly different in the wild type and the *cagI* mutant. Control immunoblots showed that the outer membrane-associated proteins CagX and AlpB were present only in the membrane fraction, and were not extracted by triton X-100, whereas the inner membrane-associated protein RecA was substantially extracted from the membrane fraction. As an independent method for membrane separation, we used isopycnic sucrose density gradient centrifugation of resuspended total membrane fractions. Immunoblot analysis of individual fractions from the sucrose gradients revealed two different membrane-associated pools of both CagI and CagL (Fig. 5B). Control immunoblots showed that only the high density fractions contained outer membrane-associated proteins, whereas the cytoplasmic membrane-associated protein RecA was present in several fractions.

While these results suggested the presence of separate CagI and CagL pools in different localizations in the bacterial cell, they did not reveal a clear association with the outer membrane or the bacterial surface. To obtain evidence for a possible localization at the surface, we performed a limited proteolytic digestion of intact bacterial cells with proteinase K. Protease accessibility of CagI and CagL was assessed by immunoblotting of equal amounts of untreated and proteinase K-treated cells. The amounts of both CagI and (to a lesser extent) CagL were significantly reduced by proteinase K treatment, whereas the cytoplasmic or cytoplasmic membrane-associated protein RecA was not digested, and the outer membrane protein AlpB was digested almost completely (Fig. 5C). Taken together, these data indicate that both CagI and CagL are partially localized at the bacterial surface, but that considerable pools of both proteins exist in a non-surface localization as well.

### CagI Interacts with CagL

**Figure 6:**
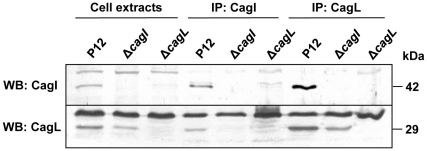
CagI interacts with CagL. Cell extracts of the wild-type strain P12 and its isogenic *cagI* (pWS327) and *cagL* mutants were subjected to immunoprecipitation (IP) with either anti-CagI or anti-CagL antisera. Cell extracts and immunoprecipitates were analysed by immunoblotting with anti-CagI and anti-CagL antisera.

The influence of CagX, CagY and CagL on CagI protein levels, and of CagI on CagL protein levels, suggested that corresponding protein-protein interactions might exist. To determine whether CagX and/or CagY interact with CagI, we performed immunoprecipitation experiments from *H. pylori* cell extracts with polyclonal CagX and CagY antisera, as described previously [24]. Western blot analysis of the precipitated proteins showed a co-precipitation of CagX with CagY and vice versa, but we were unable to detect CagI in the precipitation fractions (data not shown). To determine whether CagI interacts with CagL, we used the polyclonal CagI antiserum for immunoprecipitation experiments. Immunoblot analysis of the precipitated proteins showed that CagI was successfully precipitated, and that CagL was indeed co-precipitated with CagI (Fig. 6). In a control immunoprecipitation from the *cagI* mutant, CagL was not co-precipitated, demonstrating the specificity of the immunoprecipitation. To confirm this result, we performed a reverse immunoprecipitation using the polyclonal CagL antiserum AK271, which precipitated CagL together with a cross-reacting protein (Fig. 6). Consistent with the results described above, CagI was co-precipitated with CagL from an *H. pylori* P12 wild-type lysate. Again, immunoprecipitation from a lysate of the P12Δ*cagL* mutant did not result in precipitation of CagI. Taken together, these data demonstrate that CagI and CagL form a complex in *H. pylori* cells.

## Discussion

Type IV secretion systems are known as a highly versatile group of macromolecule transporters [27], [28], and this versatility is also reflected in the variation of their respective components. Many well-characterized type IV secretion systems including some conjugation systems are composed of conserved essential components. In contrast, more divergent systems such as the Icm/Dot (type IVb) system of *Legionella pneumophila*, or the Cag system of *H. pylori*, usually include additional components with as yet unknown functions. The Cag system probably contains functional analogues to all VirB proteins and to VirD4 [8], but also additional essential components that are unique to this system. These additional components include two proteins (Cagδ/Cag3 and CagM) which have been shown to take part in an outer membrane-associated subcomplex [24], [29], two predicted inner membrane proteins (CagU and CagH), and one protein (CagD) with a possible surface localization [30]. Using careful mutation and complementation studies, we show here that CagI is another essential component of the Cag type IV secretion apparatus. Furthermore, we show that CagI interacts with CagL, an essential Cag secretion apparatus component that, like CagI, has been shown to bind to integrin receptors on the target cell surface [13]. A recent study, which also included complementation of *cagI* mutants [20], reached the same conclusions.

The chromosomal region containing the *cagI* gene seems to be organized as an operon of five genes (*cagF*, *cagG*, *cagH*, *cagI* and *cagL*) [19], with the latter three open reading frames overlapping each other, indicating that there is a tight coupling of *cagH*, *cagI* and *cagL* gene expression. Although we found effects of *cagG* and *cagH* deletion on CagI and CagL protein levels, and of *cagI* deletion on CagL protein levels, *cagF* deletion did not seem to have much influence on the levels of either protein, arguing against simple transcriptional effects. This is also supported by our *cagL* transcript quantification data showing only minor differences between the wild-type strain and different *cagI* mutants, and by complementation of the *cagI* deletion mutants which restored CagL production without changing *cagL* transcript levels. Although we cannot exclude that translation of *cagL* transcripts or other post-transcriptional processes are less effective in the *cagI* deletion mutants, it is likely that the presence of the CagI protein and its interaction with CagL accounts at least partly for maintenance of CagL wild-type levels. A surprising finding was that the complete marker-free deletion of *cagI* (using plasmid pWS327) did not influence CagL protein levels. Since *cagL* transcript levels were increased in this mutant, the most likely explanation is that a combination of higher *cagL* transcription and increased CagL degradation due to the lacking interaction with CagI resulted in almost wild-type levels of the CagL protein. It is not clear which effect was responsible for the differing phenotype reported previously for the *cagI* mutant in strain 26695 [16], but reconstruction of 26695 *cagI* deletion mutants resulted in the same phenotypes as described here (data not shown), arguing against strain-specific differences.

It is reasonable to assume that the genetic organization of *cagG*, *cagH*, *cagI* and *cagL* also reflects a functional connection between the gene products. Previous yeast two-hybrid screening procedures [25], [31] detected interactions between CagI and several other Cag proteins including CagG, but none of them was confirmed except an interaction between recombinant CagI and CagZ fusion proteins *in vitro* [31]. We show here that the native CagI and CagL proteins take part in a protein complex in *H. pylori* cells, as also found in a recent study where immunoprecipitation of CagL from *H. pylori* lysates resulted in coprecipitation of CagI and CagH [20]. CagG, CagH, CagI, and CagL are all acidic proteins with predicted isoelectric points between 4 and 6. CagG, CagI and CagL contain N-terminal signal sequences and would thus be supposed to be transported to the periplasm, from where the latter two might reach the bacterial surface, as would be expected for integrin ligands. In contrast, CagH is most likely an integral cytoplasmic membrane protein, according to topology predictions [24]. An interesting feature of CagI is that its C-terminal amino acids (SKVIVK) are almost identical to the C-terminal amino acids of CagL (SK(I/V)IVK) and CagH (TKIIVK); the latter two proteins also show a weak overall sequence similarity between each other (data not shown). Intriguingly, these C-terminal motifs have been shown to contribute to the function of these proteins [20], raising the possibility that they represent binding motifs for a common interaction partner of all three proteins.

Given that CagI and CagL were found to interact by co-immunoprecipitation, it is worth noting that the two proteins were not distributed equally in bacterial cell fractions. The majority of CagL was found in a soluble fraction, which also contained CagI, but more CagI seemed to be associated with the membrane fractions under the conditions used. Due to their N-terminal signal sequences, the most likely localization of the soluble pools of both proteins is in the periplasm. The membrane-associated pools of CagI and CagL showed a similar distribution in sucrose density gradient fractions, although CagI was more readily extracted from a total membrane fraction with triton X-100. However, as described previously [24], [26], it is not possible to completely separate cytoplasmic and outer membrane proteins of *H. pylori* using such standard procedures. Therefore, a clear assignment of the membrane pools to cytoplasmic membrane or outer membrane-derived vesicles, respectively, is not possible at this point, but the protease digestion experiments strongly suggest a partial surface exposition of the CagI protein. CagI showed a higher susceptibility for surface proteinase K digestion than CagL, but since CagL has been localized on surface appendages formed by the Cag secretion apparatus [13] and probably functions as a pilus-associated adhesin binding to integrin receptors [15], we would expect that CagL is also surface-exposed. It is not clear at this point whether CagI localizes to type IV secretion apparatus pili as well, and if so, whether the interaction between CagI and CagL takes place there, or if there is only an indirect interaction, mediated by a common interaction partner of both proteins. Unfortunately, possibly due to the cross-reactions of the CagI antiserum, we have so far been unable to localize CagI at the bacterial surface by immunofluorescence. Thus, it remains to be shown whether CagI is really associated with the pilus-like type IV surface appendages.

The decreased CagI levels in mutants lacking components of the putative type IV secretion apparatus core complex (Δ*cagV*, Δ*cagW*, Δ*cagX*, Δ*cagY*, and possibly Δ*cagE*), or in mutants lacking components of the putative outer membrane-associated subcomplex (Δ*cagδ*, Δ*cagM*, Δ*cagX*), might be taken as an indication that corresponding protein-protein interactions exist. However, since we were unable to detect any interaction between CagI (or CagL) and CagX or CagY by immunoprecipitation (data not shown), an alternative possibility is that wild-type CagI levels reflect correct protein localization, implying that CagI is transported by the secretion apparatus itself. In this scenario, an incompletely assembled secretion apparatus would result in failure of CagI to reach its destination, to form the putative CagI-CagL complex, and, as a consequence, in protein degradation. CagI and CagL would thus represent “late” components in secretion apparatus assembly, an assumption that is also supported by the observation that type IV secretion pili are not produced in the absence of CagI or CagL [20]. Further studies are required to elucidate the order in which the different Cag proteins assemble into a functional secretion or translocation complex.

In conclusion, we have demonstrated that CagI is an essential component of the Cag type IV secretion apparatus. Dependence of CagI protein levels on multiple *cag* genes and its interaction with CagL suggest that these two proteins take part in a subcomplex at the bacterial surface which might, possibly together with further components such as CagY, tether the secretion apparatus to integrin receptors to initiate translocation of CagA into host cells.

## Materials and Methods

### Bacterial Strains, Cell Lines and Transformation

*H. pylori* strains were grown on GC agar plates (Difco) supplemented with vitamin mix (1%), horse serum (8%), vancomycin (10 mg/l), trimethoprim (5 mg/l), and nystatin (1 mg/l) (serum plates), and incubated for 16 to 60 h in a microaerobic atmosphere (85% N_2_, 10% CO_2_, 5% O_2_) at 37°C. *E. coli* strains Top10 (Invitrogen) and DH5α (BRL) were grown on Luria-Bertani (LB) agar plates or in LB liquid medium [32] supplemented with ampicillin (100 mg/l), chloramphenicol (30 mg/l), or kanamycin (40 mg/l), as appropriate. AGS epithelial cells (obtained from ATCC; number CRL-1739) were cultivated under standard conditions as described previously [33]. For the generation of isogenic mutants in *H. pylori* strain P12, the corresponding plasmids (Table 1) were introduced by natural transformation, as described [34]. *H. pylori* transformants were selected on serum agar plates containing 6 mg/l chloramphenicol or 8 mg/l kanamycin.

### Plasmid Constructions

Standard cloning and DNA analysis procedures were performed according to [32]. Plasmid DNA was purified from *E. coli* by the boiling procedure and *E. coli* cells for electroporation were prepared according to the protocol recommended for the Gene Pulser (BioRad). Amplification of DNA fragments by polymerase chain reaction (PCR) was performed as described [34]. The *cagI* deletion plasmids pWS320, pWS325 and pWS326 were based on inverse PCR amplification of a gene library plasmid used for sequencing of strain P12 [35] with primers WS418 (5′-ACCGGTCGAC TAAAAAACAT TTCATATCTC-3′) and WS419 (5′-ACCGGTCGAC GGATCCGACA ACGCTCAATA CATCG-3′). The PCR product, which contained thus *cagI* flanking regions and the pSMART-HCKan cloning vector, was either digested with *Sal*I and religated for plasmid pWS326, or digested with *Bam*HI and *Sal*I and subsequently ligated with a chloramphenicol resistance cassette to obtain plasmid pWS325, or with an *rpsL*-*erm* cassette to obtain plasmid pWS320. The precise deletion construct pWS327 was obtained by inverse PCR from the same gene library plasmid using the 5′-phosphorylated primers WS420 (5′-CATATCTCTT TCCTCACTTC-3′) and WS421 (5′-AAAACACTCG TGAAAAATAC C-3′), and blunt-end religation of the PCR product. For complementation of the *cagI* mutants, plasmid pWS322 was constructed by PCR amplification of the *cagI* gene using primers WS311 (5′-GGACTAGTGA AGTGAGGAAA GAGATGTG-3′) and WS308 (5′-ACCGCTCGAG TCATTTGACA ATAACTTTAG-3′), and cloning the PCR product via *Spe*I and *Xho*I into pWS241 [36], a derivative of the chromosomal *recA* integration vector pJP99 [37], which enables, after integration into the *H. pylori recA* locus, expression of cloned genes under the control of the *cagA* promoter.

### Antisera and Immunoblotting

The antiserum against CagI was generated by immunization of a rabbit with a synthetic peptide comprising 19 C-terminal amino acids of CagI (amino acids 350–368; H_2_N-(C)NLEKKADLWEEQLKLERET-COOH) coupled to KLH, as described previously [24]. Rabbit polyclonal antisera against CagL, CagX, CagA, RecA, and AlpB have been described previously [10], [24], [38]. Sodium dodecyl sulfate-polyacrylamide gel electrophoresis (SDS-PAGE) and Western blotting was performed as described [16]. For the development of immunoblots, polyvinylidene difluoride (PVDF) filters were blocked with 5% non-fat milk powder in TBS (50 mM Tris-HCl, pH 7.5, 150 mM NaCl), 0.1% (v/v) Tween 20, and incubated with the respective antisera at a dilution of 1∶1000–1∶5000. Alkaline phosphatase-conjugated protein A or horseradish peroxidase-conjugated anti-rabbit IgG antiserum was used to visualize bound antibody.

### Tyrosine Phosphorylation Assay and Determination of IL-8 Secretion

Standard infections of AGS cells with *H. pylori* strains and subsequent preparations for phosphotyrosine immunoblotting were performed as described previously [33]. Briefly, cells were infected with bacteria at a multiplicity of infection of 100 for 4 h at 37°C, washed three times and suspended in PBS containing 1 mM EDTA, 1 mM Na_3_VO_4_, 1 mM PMSF, 10 µg/ml leupeptin, 10 µg/ml pepstatin. Cells with adherent bacteria were collected by centrifugation and resuspended in sample solution. Tyrosine-phosphorylated proteins were analyzed by immunoblotting with the phosphotyrosine antiserum PY99 (Santa Cruz Biotechnologies). The production of IL-8 by AGS cells after infection with *H. pylori* strains for 4h was determined from cell supernatants by a sandwich ELISA as described [16].

### RNA Isolation

Total RNA was isolated from *H. pylori* strains grown in liquid cultures for 16–24 h by the hot phenol method [19]. Briefly, bacterial cells were mixed with a stop solution containing 95% ethanol and 5% phenol and collected by centrifugation. Pellets were resuspended in 600 µl TE (pH 8.0) containing 0.5 mg/ml lysozyme; suspensions were mixed with 60 µl 10% SDS, incubated at 64°C for 2 min, mixed with 66 µl 1M sodium acetate (pH 5.2), and extracted with 750 µl phenol at 64°C. Traces of phenol were removed from the aqueous phases by extraction with 750 µl chloroform and centrifugation at 12000 × g for 15 min. RNA was precipitated from the aqueous phases with ethanol/sodium acetate (pH 6.5), redissolved in diethylpyrocarbonate-treated water, and treated with RNase-free DNase I (Fermentas). Total RNA was analyzed on nondenaturing 1.0% agarose gels and quantified on a NanoDrop ND 1000 spectrophotometer (Thermo Scientific).

### cDNA Synthesis and qPCR

For cDNA synthesis, we used the First Strand cDNA Synthesis Kit (Fermentas) with 1–5 µg total RNA and random hexamer primers. Reverse transcription was carried out at 25°C for 5 min and at 37°C for 60 min, and the reaction was terminated by heating to 70°C for 5 min. Primers for qPCR were designed to amplify 90 bp to 150 bp regions of the *cagL* gene as well as 16S-rRNA (internal control). Reaction mixes containing a master mix with SYBR green (Invitrogen, Hamburg, Germany) and each set of primers were added to a 96-well plate together with diluted cDNA samples at a final volume of 25 µl per well. Nuclease-free water was used as negative control. Samples were incubated in an ABI-Prism SDS7000 (Applied Biosystems, Darmstadt, Germany) for 43 cycles (30 s at 95°C, 30 s at 59°C, and 30 s at 72°C). Transcript levels were quantified by the comparative C_T_ (cycle threshold) method and normalized to 16S-rRNA levels in each sample. The relative abundance of each transcript was calculated using the 2^-ΔCT^ formula.

### Bacterial Cell Fractionation

Bacterial cells were fractionated as described previously [26], with minor modifications. Briefly, *H. pylori* cells were grown in Brucella broth for 24–48 h, then harvested, washed and resuspended in preparation buffer (10 mM Tris-HCl, pH 8.0, 1 mM PMSF, 1 µM leupeptin, 1 µM pepstatin). Bacteria were lysed by ultrasonication, and the lysate was centrifuged for 10 min at 7000 × g to remove unbroken cells and cell debris. The supernatant was collected and separated by ultracentrifugation (60 min, 230000 × g) into soluble (cytoplasmic and periplasmic) and total membrane fractions. Proteins in the soluble fractions were concentrated by chloroform-methanol precipitation [39], while the membrane fractions were washed with and resuspended in preparation buffer. For differential extraction, triton X-100 was added to the membrane suspension to a final concentration of 1% (wt/vol), and the mixture was incubated on ice for 30 min and fractionated by ultracentrifugation (45 min, 230000 × g). Sucrose density gradients were prepared by layering solutions containing decreasing sucrose concentrations (60% (w/v) to 25% (w/v)) in triethanolamine buffer (50 mM triethanolamine, pH 7.5; 1 mM EDTA) upon one another in SW41 rotor tubes, followed by overnight equilibration at 4°C. Total membranes resuspended in triethanolamine buffer were layered on top of the gradients, and the tubes were centrifuged at 4°C (18 h, 270000 × g). Fractions were collected from the density gradients, and proteins were concentrated by precipitation as above.

### Limited Proteinase K Digestion

For limited proteinase K digestion, bacteria were grown on agar plates for 24 hours, harvested and washed in PBS, and resuspended at a density of 6 × 10^7^ cells per ml in proteinase K buffer (50 mM Tris-HCl, pH 7.4; 150 mM NaCl, 1 mM CaCl_2_, 1 mM MgCl_2_, 1 mM KCl) containing 1 mg/ml proteinase K (Sigma). This suspension was incubated at room temperature for 30 min. To stop the reaction, PMSF was added to a final concentration of 1 mM. Bacteria were collected by centrifugation at 5000 × g for 20 min at 4°C. Pellets were resuspended in radioimmunoprecipitation (RIPA) buffer (50 mM Tris-HCl, pH 8.0, 150 mM NaCl, 1 mM EDTA, 1% Nonidet P-40, 0.25% sodium deoxycholate, 1 mM PMSF, 10 µg/ml leupeptin, 10 µg/ml pepstatin) and mixed with SDS-PAGE sample solution.

### Immunoprecipitation

Bacteria grown on agar plates were suspended in PBS and washed twice. An amount of 5 × 10^10^ bacteria was resuspended in radioimmunoprecipitation (RIPA) buffer (50 mM Tris-HCl, pH 8.0, 150 mM NaCl, 1 mM EDTA, 1% Nonidet P-40, 0.25% sodium deoxycholate, 1 mM PMSF, 10 µg/ml leupeptin, 10 µg/ml pepstatin) and the cells were lysed by sonication. Unbroken cells were removed by centrifugation for 10 min at 10000 × g. To remove unspecifically interacting proteins, the lysates were incubated with prewashed protein G-agarose (Roche Diagnostics) for 2h at 4°C, and then centrifuged. To the supernatants, 5 µl of the appropriate polyclonal antisera was added, and samples were incubated for 3h at 4°C. Then, 50 µl of prewashed protein G-agarose was added and samples were incubated at 4°C for additional 2h. After three washing steps with RIPA buffer, proteins were eluted with 100 mM glycine, pH 2.7, or by boiling in SDS-PAGE sample solution.
